# Population Pharmacokinetics Model of Cyclosporin A in Children and Young Adult Renal Transplant Patients: Focus on Haemoglobin Contribution to Exposure Variability

**DOI:** 10.3390/pharmaceutics18010099

**Published:** 2026-01-12

**Authors:** Maša Roganović, Mirjana Cvetković, Ivana Gojković, Brankica Spasojević, Marija Jovanović, Branislava Miljković, Katarina Vučićević

**Affiliations:** 1 Department of Pharmacokinetics and Clinical Pharmacy, Faculty of Pharmacy, University of Belgrade, 11221 Belgrade, Serbia; 2 Department of Nephrology, Dialysis, and Transplantation, University Children’s Hospital, 11000 Belgrade, Serbia; 3 Faculty of Medicine, University of Belgrade, 11000 Belgrade, Serbia

**Keywords:** cyclosporine, population pharmacokinetics, haemoglobin, TDM, NONMEM

## Abstract

**Background/Objectives**: Cyclosporine A (CsA) is a key immunosuppressant in post-transplantation therapy protocol characterized by large interindividual and intraindividual pharmacokinetic (PK) variability and a narrow therapeutic range necessitating therapeutic drug monitoring (TDM) to prevent graft rejection and minimize side effects. TDM data can be used for developing PK models with the objective of identification and quantification of variability factors that contribute to the differences in CsA concentrations. **Methods**: Retrospectively collected data from medical records of 58 patients (children and young adults) regarding CsA blood concentrations, concomitant medications, and laboratory findings of significance were used for the population PK model development in NONMEM^®^ (version 7.5) with first-order conditional estimation method with interaction (FOCE-I). Simulation of the concentrations and area under the curve (AUC) was performed in the web application e-campsis^®^. RStudio (version 4.5.0) was used for the purpose of descriptive statistics analysis and graphs plotting. **Results**: A one-compartment model with first-order absorption and elimination best described the data. Value of clearance (CL/F) was estimated to be 15 L/h, and volume of distribution (V/F) was 71.1 L for a typical patient weighing 40 kg. Interindividual variability (IIV) on CL/F and V/F was 34.91% and 43.05%, respectively. Interoccasional variability (IOV) was 12.25%. Body weight (WT) was introduced allometrically on CL/F and V/F, with the estimated exponent of 0.89 for CL/F and 1 (fixed) for V/F. According to the final model, CL/F decreases with increasing haemoglobin (HGB) value. A difference of almost 22.5% in CL/F was observed among patients’ HGB values reported in the study. **Conclusions**: Our findings indicate that HGB levels significantly influence CsA PK, particularly minimum concentration (C_min_), highlighting the importance of regular HGB levels monitoring together with CsA levels.

## 1. Introduction

Cyclosporine A (CsA) is an immunosuppressive drug that belongs to the class of calcineurin inhibitors (CNIs) [1]. In combination with other immunosuppressive drugs, CsA forms an essential part of post-transplant therapy protocol aimed at preventing graft rejection after solid organ transplantation. Although tacrolimus has largely replaced CsA in solid organ transplantation due to lower rejection rates and better long-term outcomes, CsA remains clinically relevant in patients who develop adverse effects such as post-transplant diabetes mellitus and those at higher risk of BK polyomavirus infection [2,3]. It shows marked interindividual and intraindividual pharmacokinetic (PK) variability and has a narrow therapeutic index [4,5,6]. The absorption and elimination of CsA are highly variable: bioavailability may range from as low as 5% to nearly 90%, and terminal elimination half-life varies from 6.3 to 20.4 h depending on patient-specific factors such as comorbidities and age [7]. Such PK characteristics necessitate regular therapeutic drug monitoring (TDM) to ensure optimal therapeutic exposure and prevent graft rejection [8]. Moreover, its pronounced PK variability and complex absorption mechanisms continue to make CsA an important subject of PK research. Integrating TDM data with population pharmacokinetic (PopPK) modelling can further enhance understanding of the covariates influencing CsA disposition, enabling dose individualization to maximize efficacy and minimize toxicity [9,10,11]. The side effects of CsA can be serious and include hypertension, nephrotoxicity, an increased risk of malignancy, and neurotoxicity [12,13]. These concerns are particularly pronounced in paediatric patients, whose physiological and biochemical characteristics differ substantially from those of adults. Consequently, variability in PK and pharmacodynamics may lead to a higher incidence of toxicity or therapeutic failure in this vulnerable population, underscoring the need for targeted research [14].

Although numerous PopPK studies have been conducted in both adult and paediatric populations, important knowledge gaps remain, particularly in paediatric population [9,10,15,16]. Research in adults far outweighs that in children, largely due to ethical and logistical challenges inherent to paediatric studies. Moreover, existing studies often involve heterogeneous patient populations, including renal, hepatic, pulmonary, cardiac, and hematopoietic stem cell transplant recipients, making cross-study comparisons difficult due to differences in physiology, comorbidities, and immunosuppressive regimens. The number of studies in paediatric population is still relatively small. TDM practices for CsA also vary substantially among transplant centres. Some institutions measure trough concentrations (C_0_, in sample taken immediately before the morning dose), while other centres focus on post-dose concentration (C_2_, approximately 2 h after the morning dose), with each applying different target ranges depending on the transplanted organ, biological matrix (most commonly whole blood), and institutional protocols. These inconsistencies reflect the lack of consensus in the literature. Furthermore, although numerous covariates have been associated with CsA PK variability, their relative clinical significance remains uncertain. Specific covariates such as genetic polymorphism and biomarkers related to red blood cells other than haematocrit have not been consistently applied across the models, which highlights a critical need for investigation into these less frequently tested variables.

Therefore, the aim of this study was to develop a PopPK model of CsA in paediatric and young adult renal transplant recipients, with the goal of improving clinical practice through individualized dosing strategies based on identified and quantified covariate effects.

## 2. Materials and Methods

### 2.1. Patient Population and Study Design

The study was conducted in the University Children’s Hospital, which is currently the only centre in the Republic of Serbia performing renal transplantation in children. The study population included children and young adults who underwent kidney transplantation either at the University Children’s Hospital or abroad, but whose post-transplant follow-up was carried out at this institution. All patients who received CsA as part of their immunosuppressive regimen were included in the study, regardless of whether their therapy was subsequently switched to tacrolimus. This was a retrospective study based on data available from patients’ medical history. The collected data included CsA dosage regimens, CsA blood concentrations, concomitant medications, and relevant laboratory parameters in kidney transplant recipients. For some patients, pharmacogenetic data were also available, including polymorphisms in *CYP3A4*, *CYP3A5*, *ABCB1*, and *POR* genes. The local ethics committee gave permission for this retrospective study (Ethical Approval No. 16/223, obtained on 7 October 2025). All patients received the same immunosuppressive regimen consisting of CsA, corticosteroid, and mycophenolate acid. CsA was initially administered at a dose of 5 mg/kg/day divided into two or three daily doses. The dose was adjusted to the following target whole-blood levels: C_0_ 150–200 ng/mL and C_2_ 1200–1400 ng/mL during the first post-transplant month; C_0_ 100–150 ng/mL and C_2_ 800–1200 ng/mL during months two and three; and, thereafter, C_0_ 100–130 ng/mL and C_2_ 800–1000 ng/mL depending on the patient’s clinical condition.

### 2.2. Sampling and Cyclosporin A Concentration Measurement

CsA concentrations were measured in whole-blood samples using chemiluminescent microparticle immunoassays developed by Abbott (Abbott Park, IL, USA). The lower limit of quantification of the assay is 30 ng/mL based on functional sensitivity, and the upper limit of quantification is 1500 ng/mL, with imprecision estimated at less or equal to 15% coefficient of variation (CV). Concentrations were measured in samples corresponding to C_0_ and C_2_, collected after at least three consecutive days of unchanged dosing regimen to ensure that steady-state was reached. The majority of the concentrations in the dataset were obtained approximately one year post-transplantation. For one patient, concentration measurements were collected approximately two years post-transplantation due to unavailable dosing history of CsA during the first-year post-transplant period.

### 2.3. Population Pharmacokinetic Modelling

PopPK analysis was performed using nonlinear mixed effects modelling with NONMEM^®^ software (version 7.5.0, ICON Development Solutions Inc., Dublin, Ireland) with first-order conditional estimation method with interaction (FOCE-I). NONMEM outputs were handled in R (version 4.5.1, R Foundation for Statistical Computing, Vienna, Austria) within RStudio (version 4.5.0, Posit Software, Boston, MA, USA) for graphical diagnostics and model evaluation. A one-compartment and two-compartment model with first-order elimination were tested. Model selection and comparison were based on a decrease in the objective function value (ΔOFV), as well as on visual inspection goodness of fit (GOF) plots. Additive, exponential, and proportional models were tested to explain interindividual variability (IIV). Residual unexplained variability was explored using additive, proportional, and combined error models.

Initial model development attempted to estimate the absorption rate constant (Ka); however, sparse sampling in the absorption phase did not support reliable estimation of this parameter, leading to model instability and imprecise estimates. Consequently, Ka was fixed during further modelling steps to ensure model identifiability and robustness and allowed reliable estimation of clearance and covariate effects. A similar approach was applied by Xue et al., who fixed Ka at 1.25 h^−1^ [17]. A sensitivity analysis was performed by varying Ka within a plausible range. Interoccasional variability (IOV) was implemented on CL/F. Every pair of concentrations measured on the same day, or a single concentration measured on a given day, was treated as a separate occasion. The correlation between CL/F and V/F was also examined.

Covariate model building was performed using the stepwise covariate modelling (SCM) procedure. Continuous covariates that were tested included age (AGE), body weight (WT), height (HT), albumin levels (ALB), cold and warm ischemia time (COLDIH, WARMIH, respectively), haematocrit (HCT), haemoglobin (HGB), serum creatinine levels (CREAT), and creatinine clearance (CLCR) calculated using the Schwartz formula (Equation (1)):

(1) $$CLCR=\frac{\left(k\cdot HT\right)}{CREAT}$$

where k is coefficient 0.413 according to the revised Schwartz formula, also known as the bedside Schwartz formula [18]. Influence of categorical covariates was also examined: gender (GEND), type of transplanted graft—live or cadaveric transplantation (TRANS), and genetic polymorphism for *CYP3A4*, *CYP3A5*, *ABCB1*, and *POR*. The forward inclusion process was set for the significance of *p* < 0.05, and the backward elimination process was set to *p* < 0.01. Correlation between the continuous covariates was examined to assess the plausibility of the results of the SCM procedure. Additionally, covariates were considered clinically significant if the change in the parameter value was greater than 20% over the range of the usual values of the covariates [19]. Additionally, the WT effect was carefully considered. Since most of the population was paediatric, the allometric scaling model for both CL/F and V/F was also tested during model development using the equations below (Equations (2) and (3)):

(2) $$CL/F=CLpop\cdot {\left(\frac{WTi}{WTmedian}\right)}^{0.75}$$

(3) $$V/F=Vpop\cdot {\left(\frac{WTi}{WTmedian}\right)}^{1}$$

Additionally, the effect of WT on CL/F was tested using allometric scaling without fixing the exponent at 0.75 and allowing its estimation to determine whether it improved model fit, given that our population included both children and young adults. Model validation was performed using techniques of internal validation, both graphical and numerical. Graphical evaluation methods included GOF plots and variability- and prediction-corrected visual predictive checks (VPC) based on 1000 simulated samples. Additionally, a bootstrap analysis was performed using 1000 replicates.

### 2.4. Simulations

Using the web application e-campsis^®^, simulations of minimal (C_min_) and maximal (C_max_) concentrations, as well as areas under the concentration-time curve (AUC) were performed under four different scenarios utilising the developed CsA PopPK model. First, a dataset consisting of 500 girls and 500 boys was simulated in RStudio^®^ using WT distribution data from the Centers for Disease Control and Prevention growth chart for twelve-year-old boys and girls, which corresponds to the median value of age in our population [20]. This dataset was used to calculate means ± standard deviation (SD) of WT for such simulated datasets, so that WT distribution can be used in simulation in e-campsis^®^ for all four different scenarios. Each scenario included 100 patients, with different values of the covariates from the final PopPK model and two occasions per patient considered. Simulations were performed using 5 mg/kg dose, consistent with both our clinical practice and recommended maintenance dose [21].

## 3. Results

### 3.1. Patients’ Characteristics

A total of 58 patients with kidney transplants were included in the study. Most of the population were male patients (34, 58.62%). The patients’ age at transplantation ranged from 1 to 25 years, with a median of 12 years (mean 11.78 years). Most of the population were children under the age of 18 (47, 81.03%), and 11 patients (18.97%) were classified as young adults (18–25 years). A total of 30 patients had live donor transplantation, and 28 had an organ transplanted from a cadaver. Genetic polymorphism was available for 33 patients. Other demographic and clinical characteristics of patients are shown in Table 1.

### 3.2. Cyclosporine Concentrations

A total of 974 steady-state concentrations were measured in whole-blood samples taken from the patients: 471 C_0_ concentrations, 501 C_2_ concentrations, one concentration measured in a sample taken 4 h after the morning dose, and one concentration 6 h post morning dose. Statistics regarding CsA concentrations are shown in Table 2.

**Figure 1:**
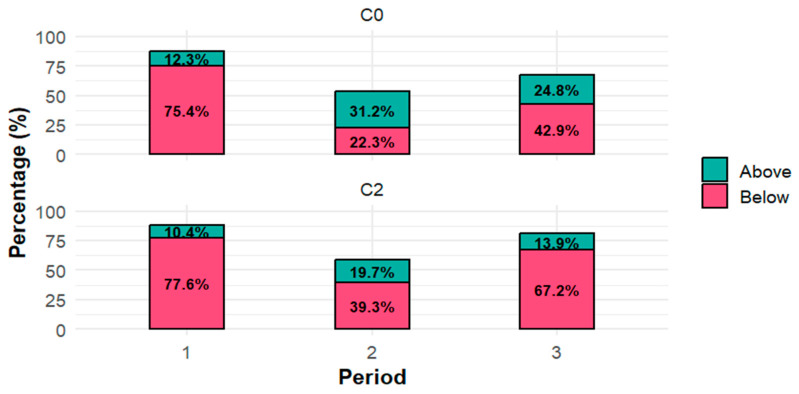
Percentage of cyclosporine A (CsA) trough (C_0_) and 2 h post-dose (C_2_) concentrations above (green) and below (red) the target concentration range across post-transplant periods. Period 1: post-transplant day (PDAY) ≤ 30; Period 2: 30 < PDAY ≤ 90; Period 3: PDAY > 90.

Figure 1 shows the percentage of C_0_ and C_2_ concentrations measured across post-transplant periods 1, 2, and 3 (corresponding to the period of PDAY ≤ 30, 30 < PDAY ≤ 90, and PDAY > 90, respectively) that are above and below the aforementioned target concentration range. The most pronounced deviation from the target concentration range was for both C_0_ and C_2_ in the first post-transplant period, with over 75% concentrations being below the desired range.

### 3.3. Population Pharmacokinetic Model

**Figure 2:**
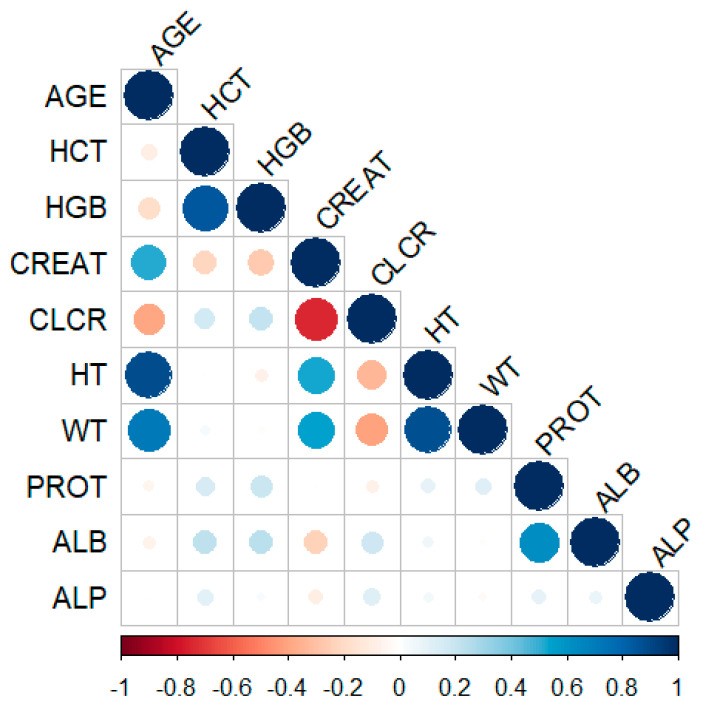
Correlation between continuous covariates. Circle size represents the strength of the correlation (larger circles indicate a stronger correlation) and colour indicates direction (blue for positive, red for negative). Colour intensity reflects the strength of correlation (darker shades indicate a stronger correlation).

The one-compartment model with first-order absorption and elimination best described the data. A base model was developed using the ADVAN2 TRANS2 subroutine and CL/F and V/F were estimated, while Ka was fixed to the value of 1.15 1/h, as it could not be reliably estimated due to limited data in the absorption phase. A sensitivity analysis was performed by varying Ka within a plausible range, demonstrating that changes in Ka had minimal impact on the primary parameter estimates, confirming that the model results are robust to the fixed Ka value. IIV was introduced using an exponential model for both parameters. IOV was kept on CL/F. The maximum number of occasions per patient was 33 over one year period. The correlation between CL/F and V/F was also accounted for in the model. After completion of the SCM procedure, the model included a linear WT effect on CL/F and V/F, and HGB, CREAT, and CLCR as covariates on CL/F. HCT, although tested, was not included in either forward inclusion or backward elimination steps. *CYP3A4* genetic polymorphism was included after forward selection step, but was not retained in the final model, and when tested manually, its effect lacked robustness, as the 95% bootstrap confidence interval included zero, likely due to the limited sample size (genotype data available for only 33 patients). Other polymorphisms were not selected during the SCM procedure in either the forward or backward steps and were therefore not tested manually. To further refine these results and assess their plausibility, a correlation analysis of continuous covariates was performed. Results from the correlation analysis are shown in Figure 2.

As CRCL and CREAT were found to be highly correlated, the final model was refined by testing each covariate separately. First, a model was evaluated with CRCL and HGB on CL/F, and WT on V/F. WT on CL/F was excluded since WT is highly correlated with HT, which is already incorporated in the calculation of CRCL. However, the relative standard error (RSE) for the CRCL effect on CL/F was extremely high (572.7%).

Next, a model including CREAT and WT on CL/F, and WT on V/F, was tested. In this case, the RSE for the CREAT effect on CL/F was much smaller (58.8%) compared to the RSE for the CRCL effect on CL/F, but still high. Nevertheless, although the effect of CREAT on CL/F was statistically significant, the magnitude of its influence was not considered clinically relevant since the change in the parameter value across the range of usual values was less than 20%, and thus the covariate was not retained.

The model was subsequently refined to include a linear effect of HGB on CL/F, and WT on both CL/F and V/F. Given that the study population was predominantly paediatric, allometric scaling was evaluated: CL/F was tested with a fixed exponent of 0.75, as well as with estimation of the exponent for CL/F (while the exponent for V/F was kept fixed at 1). WT was centred to the rounded median value of 40 kg. The latter approach yielded the most stable and robust model. This model was therefore selected as the final model. Parameter estimates are summarized in Table 3.

Below are shown Equations (4) and (5) from the final model for CL/F and V/F, where WTi and HGBi are the individual WT and individual value of HGB, respectively.

(4) $$CL/F=15\cdot {\left(\frac{WTi}{40}\right)}^{0.89}\cdot (1-0.00279\cdot \left(HGBi-120\right))$$

(5) $$V/F=71.1\cdot {\left(\frac{WTi}{40}\right)}^{1}$$

**Figure 3:**
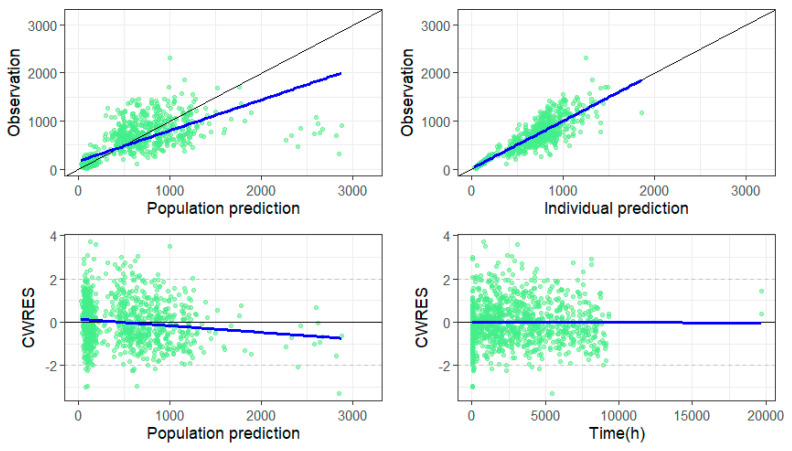
Goodness of fit plots. Observation vs. population prediction and observation vs. individual prediction graphs: dots represent individual data points, black line represents line of identity (y = x), blue line represents regression line; CWRES vs. population prediction and CWRES vs. time: black line represents line of identity (y = 0), blue line represents regression line.

**Figure 4:**
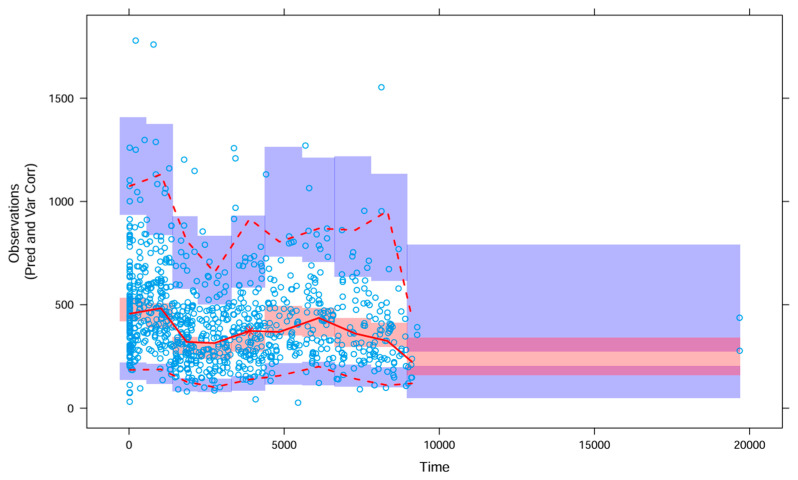
Prediction and variance corrected visual predictive check (VPC). Observed concentrations are shown as circles. Solid and dashed red lines represent median, 5th and 95th percentile of the observed data, with shaded 95% confidence intervals of the simulation-based prediction intervals for median (red) and the 5th and 95th percentiles (purple).

Model appropriateness was checked using basic GOF and variability and prediction corrected VPC (Figure 3 and Figure 4, respectively). As it can be seen, the graph of the dependent variable, i.e., observed concentrations vs. population predicted concentrations, shows reasonable distribution of points around the line of identity, while the graph of observed concentrations vs. individual predicted concentrations shows symmetrically distributed points around line of identity with an almost perfect match. The graph of conditional weighted residual (CWRES) vs. population predictions shows that the majority of CWRES lies between ±2 units of the ordinate.

The VPC (Figure 4) demonstrated adequate agreement between observed and predicted concentrations, with only a few observations falling outside the prediction intervals. Data points around 20,000 h (time since first dose) correspond to a single patient, for whom PK data were available approximately two years after therapy initiation.

### 3.4. Simulation Results

The simulation was performed based on a WT distribution for a typical 12-year-old. The mean value of WT calculated from the simulated dataset that consisted of 1000 12-year-old patients, with 50% being boys, and 50% being girls, was calculated to be 43.03 kg and SD of 9.89 kg. Four different scenarios were simulated: two groups where patients had a low value of HGB (73, taken from the data range that we observed in our real dataset) on occasions 1 and 2, and two groups with a normal value of HGB 120 (also median value of our dataset) simulated on two occasions. Simulated AUC, C_max_, and C_min_ are shown in Table 4. From the values in the table, the group with normal HGB had higher AUC, C_max_, and C_min_ on both occasions. Compared with target C_0_ and C_2_ concentration ranges (regardless of the post-transplant period), simulated concentration values are higher. The higher simulated concentrations are likely due to dosing per kg being applied to a narrower range of WT, whereas the original dataset used to develop the model included a much wider WT range (9.8–103 kg).

## 4. Discussion

We developed a CsA one-compartment model with first-order absorption and elimination in this mixed population, with children (<18 years old) being a major part of the studied population. CL/F and V/F were estimated for a typical patient with 40 kg at 15 L/h and 71.1 L, respectively, while Ka was fixed to the value of 1.15 1/h. Among the tested covariates, allometrically scaled WT on CL/F and V/F, and HGB on CL/F are retained. To our knowledge, this is the first population model to describe the influence of HGB on CsA CL/F variability in paediatric patients.

Depending on how sparse or rich the concentration data was, different models were used as a structural model for CsA in previous studies. Similar to our model, many studies used a one-compartment model [17,22,23,24,25,26,27,28]. There are also studies that used a two-compartment or even three-compartment model [4,29,30].

A study by Lukas et al. estimated CL/F to the value of 17 L/h with IIV of 27% in adult renal transplant patients, which is very close to the value we estimated. The same study estimated V/F to be 134 L, and that is larger than our estimated value of V/F [31]. Yin et al. reported V/F being 4.5 L/kg, which is again higher than the value we reported [32]. Both studies had Ka fixed to higher values (4 1/h and 2 1/h, respectively) compared to our estimate, which likely resulted in the larger estimated V/F values [31,32]. Previously published parameter values reported by Parke et al. (Ka 1.25 1/h and V/F 77.4 L), are close to our fixed Ka value and V/F estimate, supporting their plausibility [33]. However, it is important to keep in mind that some differences in disposition parameters may be related to the weight differences between children and adults.

Including maturation functions is a common practice in PopPK models, where the population studied includes children [14]. Due to the limited number of very young children (3 patients in total being under or equal to the age of 2), no maturation function was applied in our study. This is physiologically plausible, as CsA metabolism reaches near-adult levels at the age of 2, and most patients in this dataset were older than 2 years. Allometric scaling of PK parameters is based on physiological principles: CL/F typically scales with WT to the 0.75 power, reflecting the relationship between body size, organ blood flow, and metabolic capacity, while V/F scales approximately linearly with WT, reflecting tissue and body water mass [14,34]. The estimated allometric exponent for CL/F was 0.89, slightly higher than the usual 0.75, whereas CL/F increases somewhat more steeply with larger body size. This finding is physiologically plausible and supports the use of WT-based dosing in this population. A study by Kim et al. also included WT as a power function, with the difference in WT being centred on 70 kg and the estimated exponent being much smaller than ours (0.419) [35]. Fanta et al. also used allometrically scaled WT, but with fixed exponents of 0.75 and 1 for CL/F and V/F, respectively [4].

A key novel finding of this study is the identification of HGB as a significant determinant of CsA CL/F. To our knowledge, this is the first study that incorporates HGB as a covariate in the PopPK model of CsA. HGB was included as a linear covariate on CL/F. According to our model, the difference between the lowest and highest HGB levels (range 73–164 g/L) corresponds to a difference of almost 22.5% change in CL/F, with CL/F decreasing as HGB increases across the range. Simulation results showed that HGB had a consistently large effect on C_min_ on both occasions, with patients with an HGB value of 120 g/L having substantially higher C_min_ compared with those with HGB of 73 g/L (53.95% on occasion 1 and 43.89% on occasion 2). However, the impact of HGB on C_max_ and AUC was less pronounced, where patients with HGB 120 g/L again exhibited higher exposure (22.37% and 23.87% increase, respectively, on occasion 1 and 6.10% and 7.82%, respectively, on occasion 2), which is expected, since these parameters are less dependent on CL/F compared to C_min_.

This effect may be of particular clinical relevance in renal transplant patients, who frequently present lower HGB levels compared with healthy children or adults [36]. Given that even modest alterations in CsA exposure can markedly affect the risk of under- or over-immunosuppression, HGB-related variability in CL/F and consequently in C_min_, C_max_, and AUC warrants careful consideration. A possible explanation for this HGB effect on CsA CL/F is that HGB may influence CsA disposition due to erythrocyte binding; a lower HGB could reduce total CsA binding, effectively increasing CL/F. Many studies showed that HCT, another biomarker related to the erythrocyte, can have an effect on CL/F for the same reason related to the distribution of CsA [4,6,9,17,24,26,32]. Nevertheless, our results suggest that HGB may serve as a more sensitive and clinically relevant biomarker for capturing variability in CsA PK, particularly in paediatric and young adult renal transplant recipients.

This study has several limitations. Firstly, the sample size is relatively small and did not include the full paediatric age spectrum, particularly younger patients in whom hepatic enzyme ontogeny plays a more prominent role. The median age of the study population was 12 years, indicating that most patients were beyond early developmental maturation. Therefore, the observed PK variability is more likely attributable to interindividual differences in physiological and haematological parameters rather than ongoing enzyme maturation. Data were collected retrospectively, which probably affected their accuracy to some extent. Furthermore, data on genetic polymorphism were not available for all patients, which, combined with the small sample size, resulted in the model in which none of the polymorphisms were included in explaining variability in CsA PK parameters. Such downsides and challenges could be overcome with a prospective study that includes more patients and more complete data.

## 5. Conclusions

The results of this research suggest that CsA PK can be influenced by HGB levels, representing, to our knowledge, the first report of such a finding. As kidney transplant patients are prone to anaemia, CsA therapy in this population should involve careful monitoring of drug concentrations and HGB, which is routinely checked in these patients, along with other biomarkers, making this convenient for everyday practice. As shown, large changes may occur mainly in C_min_, while C_max_ and AUC changes are moderate compared with C_min_. These variations can result in significant deviations from target concentration ranges, which are crucial for effective and safe therapy in this vulnerable population.

## Figures and Tables

**Table 1:** Descriptive statistics of patients’ characteristics (*n* = 58).

Characteristics	Mean	SD	Median	Range	IQR
HT (cm)	137.21	25.20	141	78–185	36.5
WT (kg)	39.95	19.53	39.65	9.8–103	26.12
CREAT (µmol/L)	107.50	70.022	87	28–696	58.25
CLCR (mL/min/1.73 m^2^)	56.29	19.82	55.21	8.93–131.58	25.39
HGB (g/L)	118.33	14.99	120	73–164	20.25
HCT (%)	35.65	4.37	35.70	21.7–49.8	6.4
PROT (g/L)	61.29	7.026	63	30–77	11
ALB (g/L)	36.91	4.47	37	10–48	3
ALP (IU/L)	146.48	129.55	112	34–964	101.5

SD—standard deviation, IQR—interquartile range, HT—height, WT—body weight, CREAT—serum creatinine, CLCR—creatinine clearance, HGB—haemoglobin, HCT—haematocrit, PROT—total protein, ALB—albumin, ALP—alkaline phosphatase.

**Table 2:** Descriptive statistics of cyclosporine (CsA) concentrations across post-transplant periods.

	PDAY ≤ 30 (*n* = 132)	30 < PDAY ≤ 90 (*n* = 229)	PDAY > 90 (*n* = 611)
C_0_ (ng/mL)			
*n*	65	112	294
Mean (SD)	119.39 (77.54)	142.07 (68.17)	113.50 (47.42)
Range	9.00–462.00	37.20–506.60	33.50–470.00
Median	113	129.7	103.15
C_2_ (ng/mL)			
*n*	67	117	317
Mean (SD)	897.18 (376.60)	915.79 (342.29)	716.69 (243.68)
Range	317.50–1850.40	111.40–2306.00	201.60–1414.00
Median	811.15	896	684

C_0_—trough concentration, C_2_—concentration 2 h after dose, PDAY—post-transplant day, SD—standard deviation.

**Table 3:** Parameter estimates and bootstrap median of the final population pharmacokinetic (PopPK) model of cyclosporine (CsA).

Parameter (Units)	Estimated Value (%RSE)	Bootstrap Median (95% CI)
CL/F (L/h/40 kg)	15 (4.7)	14.92 (13.63–16.35)
V/F (L/40 kg)	71.1 (5.8)	70.76 (63.034–79.71)
Wp (%)	0.258 (4.3)	0.26 (0.23–0.28)
θ_ALL	0.89 (5.7)	0.89 (0.78–0.98)
θ_HGB	−0.00279 (24)	−0.0028 (−0.0041–−0.0016)
ω (CL-V)	0.136 (27.3)	0.13 (0.068–0.20)
IIV_CL_ (%)	34.91 (15.5)	33.83 (22.56–44.65)
IIV_V_ (%)	43.05 (11.8)	41.83 (31.7–52.49)
IOV_CL_ (%)	12.25 (12.5)	12.14 (8.7–15.87)

CL/F—clearance, V/F—volume of distribution, Wp—proportional error, θ_ALL—estimated allometric coefficient for CL/F, θ_HGB—haemoglobin effect on CL/F, ω (CL-V)—covariance between CL and V, IIV_CL_, IIV_V_—interindividual variability for CL and V, respectively, IOV_CL_—interoccasional variability on CL.

**Table 4:** Simulated exposure metrics.

Metric	Low HGB OCC1 *n* = 100 ^1^	Normal HGB OCC1 *n* = 100 ^1^	Low HGB OCC2 *n* = 100 ^1^	Normal HGB OCC2 *n* = 100 ^1^
AUC (ng·h/mL)	6343 (3784–12,189)	7857 (3666–13,174)	6687 (3891–14,707)	7210 (3908–15,118)
C_min_ (ng/mL)	215 (119–528)	331 (173–597)	221 (120–528)	318 (154–631)
C_max_ (ng/mL)	1851 (1093–3545)	2265 (1019–3793)	1918 (1092–4255)	2035 (1121–4340)

^1^ Median (5% Centile–95% Centile). AUC—area under the concentration–time curve, C_min_—minimal CsA concentration, C_max_—maximum CsA concentration, HGB—haemoglobin, OCC—occasion number, *n*—number of patients per group.

## Data Availability

The data that support the findings of this study are not openly accessible due to ethical restrictions.
